# Supplementary material for: Constructing graphs from genetic encodings
Source: Sci Rep. 2021 Jun 24;11:13270. doi: 10.1038/s41598-021-92577-2 (PMC8225892; doi:10.1038/s41598-021-92577-2)
Supplement: Supplementary file 1 — Supplementary Information. [file 41598_2021_92577_MOESM1_ESM.pdf]

Constructing Graphs from Genetic Encodings

Dániel L. Barabási and Dániel Czégel

Correspondence: barabasi@fas.harvard.edu

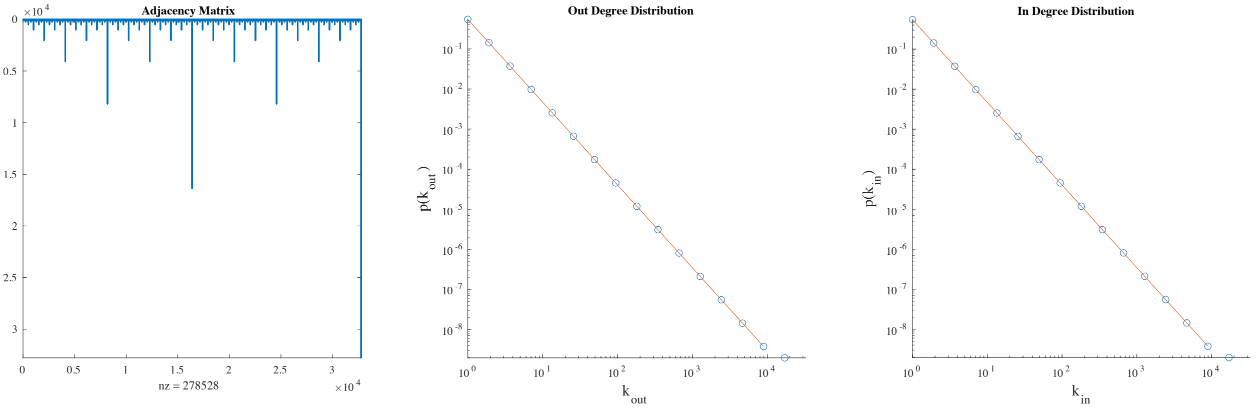

Figure S 1: **Scale-Free distributions using the connectome model.** Left: Adjacency matrix resulting from b = 15. Middle, right: Degree distributions of scale-free graph of b = 15. Both in- and out-degree distributions are best fit by $p(k) = 0.5 * k^{-1}$, as long as the outlier highest degree is excluded.

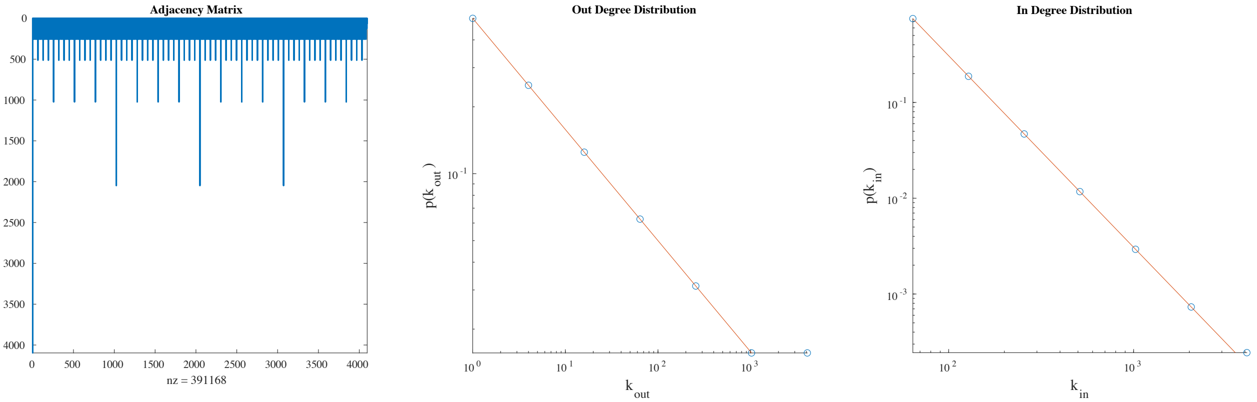

Figure S 2: **Scale Free Distribution with −2 exponent.** Left: Adjacency matrix resulting from b = 12. Middle, right: Degree distributions of scale-free graph of b = 12. While the standard model has matching in- and out-degree distribution, this alternative model has an out-degree distribution is best fit by $p_{out} \sim k^{-0.5}$, and in-degree distribution is best fit by $p_{in} \sim k^{-2}$.

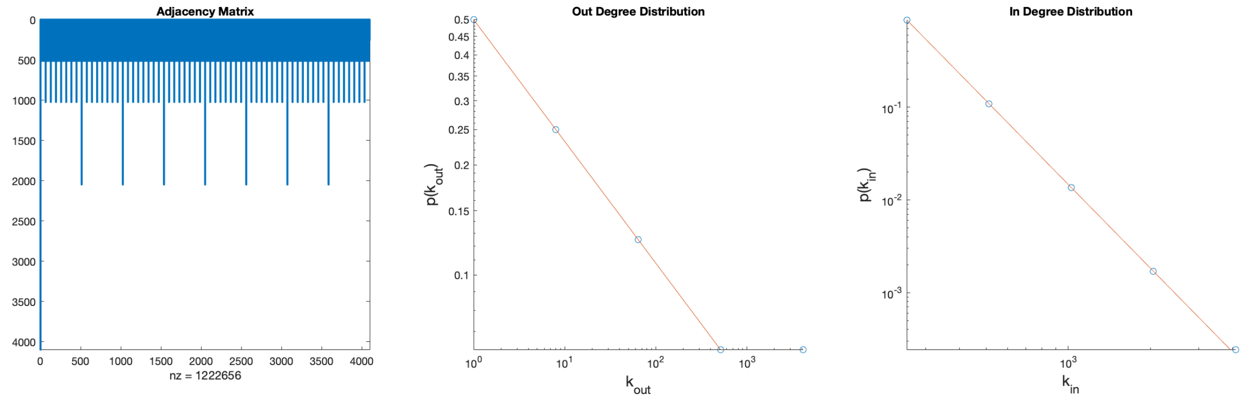

Figure S 3: **Scale Free Distribution with -3 exponent.** Left: Adjacency matrix resulting from b = 12. Middle, right: Degree distributions of scale-free graph of b = 12. While the standard model has matching in- and out-degree distribution, this alternative model has an out-degree distribution is best fit by $p_{out} \sim k^{-1/3}$, and in-degree distribution is best fit by $p_{in} \sim k^{-3}$.

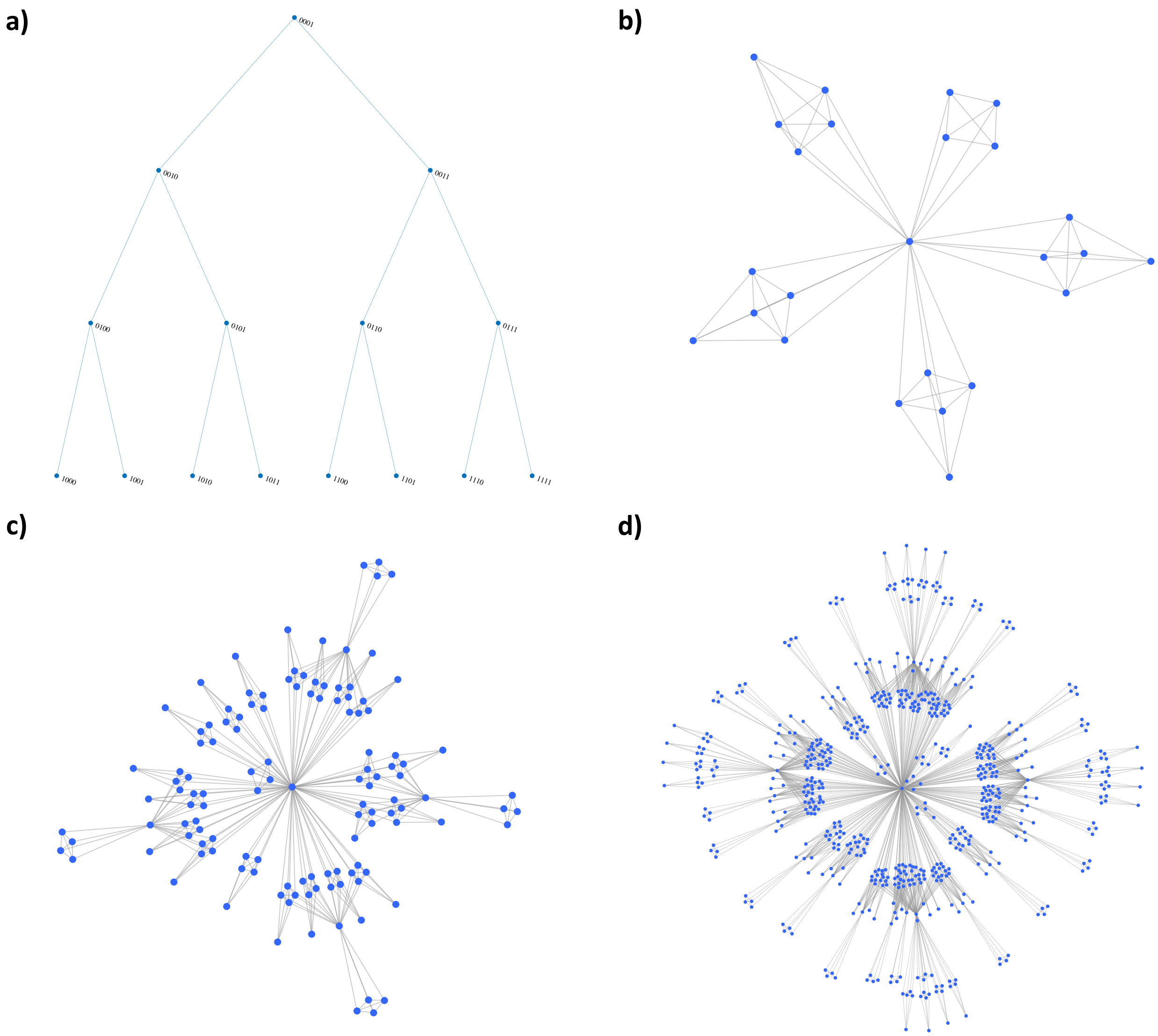

Figure S 4: **Heuristic Models for Well-Studied Networks.** **(a)** Binary tree of depth 4, with node barcodes labeled. **(b-d)** Hierarchical networks for n = 1 (b), n = 2 (c), and n = 3 (d).

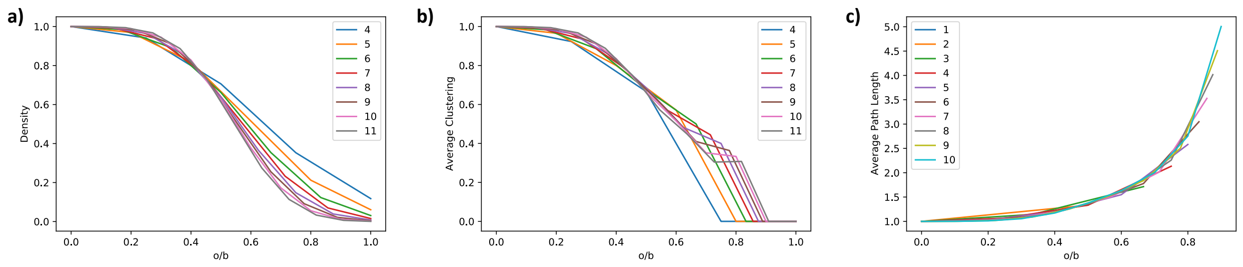

Figure S 5: **Networks Arising from Homophily Wiring Rules.** Plots of **(a)** Density, **(b)** Average Clustering, and **(c)** Average Path Length as a function of o/b (overlapping bits normalized by bit-length of identity) are shown for various system sizes (expressed as b values in legend). For all metrics the various system sizes (b values) display nearly identical scaling, with deviations attributable to small system sizes.

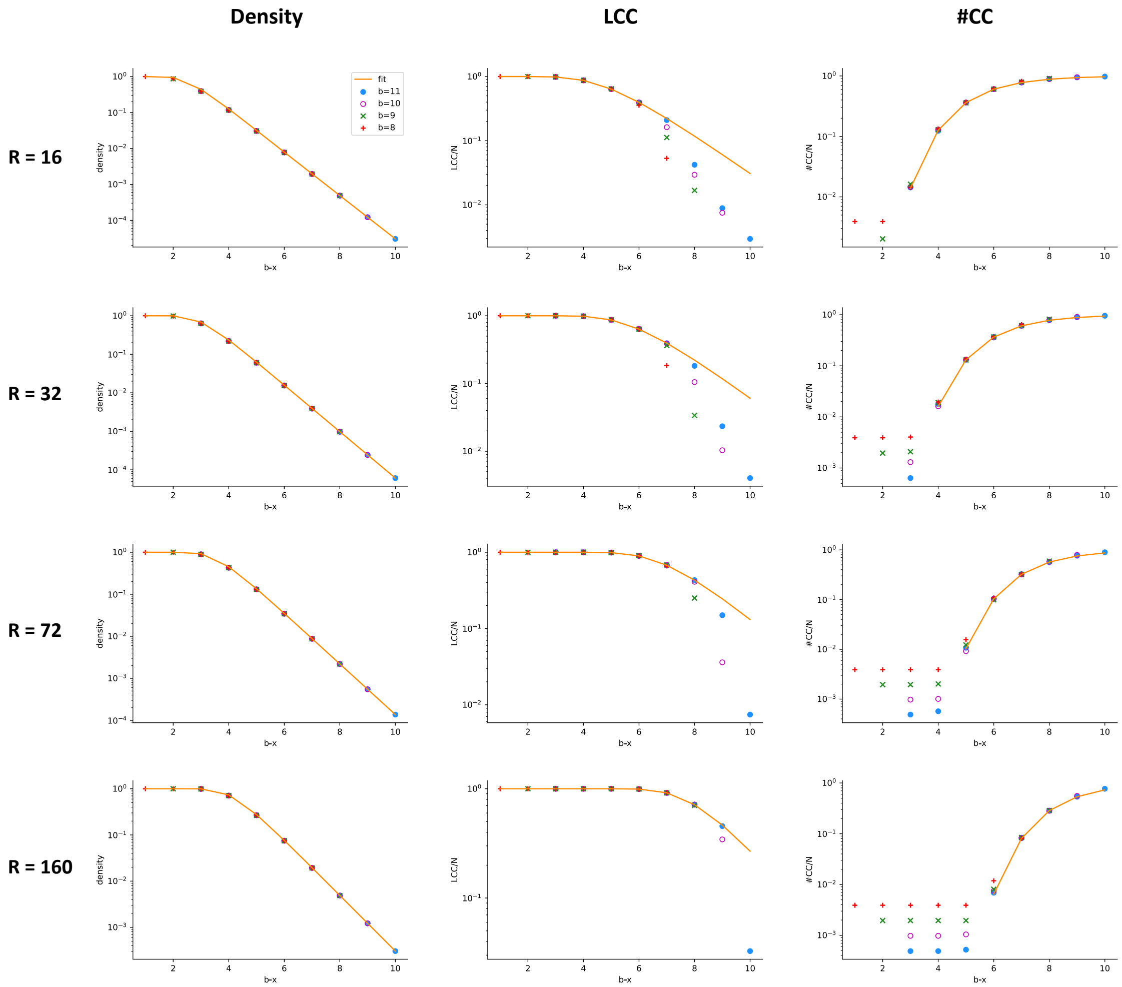

Figure S 6: **Scaling of Network Metrics with System Size.** Plots of Density, LCC and $\#CC$ are shown for four r values. In all metrics and r values, the various system sizes display nearly identical scaling, with deviations attributable to finite size arguments, as expressed in Methods 1.3.2.

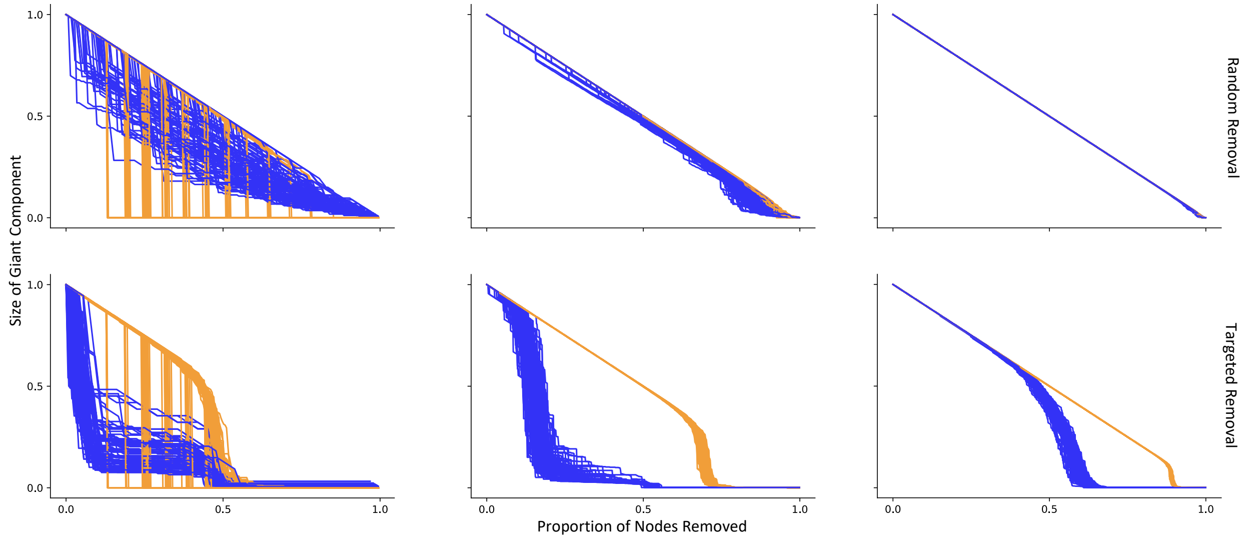

Figure S 7: **Additional Simulations for Network Robustness.** Robustness of RG networks (blue) and ER networks of equivalent density (orange), under random removal (top row) and targeted attacks (bottom row). Each line corresponds to a single simulation, for a total of 100 initialization of each RG and ER parameter set. Each column's r and x values correspond to the values set in Figure 4d-f above (left: $x = 3$, $r = 15$, middle: $x = 4$, $r = 30$, right: $x = 5$, $r = 70$). The y-axis measures the relative size of the current LCC compared to the initial giant component, and the x-axis corresponds to the proportion of nodes removed. For both y- and x-axes, the first tick corresponds to 0.5 and the second tick, where visible, corresponds to 1.
